# P05-05 Impact of pain and fear of falling in walking performance under single and dual-task conditions in women with fibromyalgia

**DOI:** 10.1093/eurpub/ckac095.072

**Published:** 2022-08-29

**Authors:** Gusi Narcis, Alvaro Murillo-Garcia, Juan Luis Leon-Llamas, Jesus Sanchez-Gomez, Santos Villafaina

**Affiliations:** Faculty of Sport Sciences, University of Extremadura, Caceres, Spain

**Keywords:** Test, fitness, dual task, pain, fibromyalgia

## Abstract

**Background:**

Fibromyalgia is characterized by stiffness and widespread pain which cause a negative impact on health-related quality of life and activities of daily living. In this regard, these activities are usually presented as dual-task situations (execution of two tasks simultaneously). Some physical fitness tests have been used under dual-task condition in order to evaluate physical fitness under a more ecological approach. It is hypothesized that physical fitness test could provide more information than physical fitness test under single-task condition. Therefore, the aim of the present study was to explore the relationship between 10m walking test performance under both single and dual- task conditions and the fear of falling and the intensity of pain in women with fibromyalgia.

**Methods:**

A total of 38 women (55.65 [9.28] years-old) participated in the study. Participants performed the 10-m walking test where they have to walk on a 10 meters straight-line as fast as they can. Moreover, the fear of falling and the intensity of pain were measured by a visual analogue scale (0-100).

**Results:**

Furthermore, the performance of 10-m walking test correlated positively with the fear of falling in both under single (rho= 0.550; p-value: > 0.001) and dual-task conditions (rho= 0.483; p-value: 0.002). However, the performance of 10m walking test only positively correlated with pain under dual-task condition (rho= 0.341; p-value: 0.031). In single task condition did not significantly correlate (rho= 0.252; p-value: 0.116).

**Conclusions:**

The performance of 10m walking test under single and dual-task conditions are correlated with the fear of falling. However, the pain intensity only significant correlated with 10m walking test performance under dual-task condition. This could indicate that pain intensity has more influence than the fear of falling on decreasing the performance of a daily living activity such as walking. However, further studies under dual-task paradigm are needed to confirm this hypothesis.

